# The Impact of Dementia on Cancer Treatment Decision-Making, Cancer Treatment, and Mortality: A Mixed Studies Review

**DOI:** 10.1093/jncics/pkab002

**Published:** 2021-01-27

**Authors:** Yaelin Caba, Kavita Dharmarajan, Christina Gillezeau, Katherine A Ornstein, Madhu Mazumdar, Naomi Alpert, Rebecca M Schwartz, Emanuela Taioli, Bian Liu

**Affiliations:** 1 Department of Population Health Science and Policy, Icahn School of Medicine at Mount Sinai, New York, NY 10029, USA; 2 Brookdale Department of Geriatrics and Palliative Medicine, Icahn School of Medicine at Mount Sinai, New York, NY, USA; 3 Department of Radiation Oncology, Icahn School of Medicine at Mount Sinai, New York, NY, USA; 4 Tisch Cancer Institute, Icahn School of Medicine at Mount Sinai, New York, NY, USA; 5 Institute for Healthcare Delivery, Icahn School of Medicine at Mount Sinai, New York, NY, USA; 6 Department of Occupational Medicine, Epidemiology and Prevention, Zucker School of Medicine at Hofstra/Northwell, Manhasset, NY, USA

## Abstract

Dementia and cancer occur commonly in older adults. Yet, little is known about the effect of dementia on cancer treatment and outcomes in patients diagnosed with cancer, and no guidelines exist. We performed a mixed studies review to assess the current knowledge and gaps on the impact of dementia on cancer treatment decision-making, cancer treatment, and mortality. A search in PubMed, Medline, and PsycINFO identified 55 studies on older adults with a dementia diagnosis before a cancer diagnosis and/or comorbid cancer and dementia published in English from January 2004 to February 2020. We described variability using range in quantitative estimates, ie, odds ratios (ORs), hazard ratios (HRs), and risk ratios (RR) when appropriate and performed narrative review of qualitative data. Patients with dementia were more likely to receive no curative treatment (including hospice or palliative care) (OR, HR, and RR range = 0.40-4.4, n = 8), while less likely to receive chemotherapy (OR and HR range = 0.11-0.68, n = 8), radiation (OR range = 0.24-0.56, n = 2), and surgery (OR range = 0.30-1.3, n = 4). Older adults with cancer and dementia had higher mortality than those with cancer alone (HR and OR range = 0.92-5.8, n = 33). Summarized findings from qualitative studies consistently revealed that clinicians, caregivers, and patients tended to prefer less aggressive care and gave higher priority to quality of life over life expectancy for those with dementia. Current practices in treatment-decision making for patients with both cancer and dementia are inconsistent. There is an urgent need for treatment guidelines for this growing patient population that considers patient and caregiver perspectives.

Between 2000 and 2050, the population of those older than 60 years will double from 11% to 22% (1). As this population ages, we expect the occurrence of aging associated comorbidities, such as cancer and dementia, to increase substantially. In the United States, the median age at the time of a cancer diagnosis is 66 years (2) and is 83.7 years at a dementia diagnosis (3). Researchers anticipate a 67% increase in cancer incidence among patients aged ≥65 years from 2010 to 2030 (4). Similarly, the number of patients with dementia worldwide is projected to increase from 50 million today to 82 million in 2030 and to 152 million in 2050 (5). A systematic review found the prevalence of older adults with cancer and dementia ranges between 0.2% and 46%, where differences are due to a variety of data collection methods and settings among individual studies (6). The co-existence of these two illnesses has serious health and economic impact on patients and their families, as well as health-care systems (7-10) As of 2018, dementia costs in the United States were $277 billion per year, surpassing the entire gross domestic product of Finland. Similarly, the cost of cancer jumped from $137.4 billion in 2010 to $147.3 billion in 2017 (2). Despite projected increasing trends of older adults living with both cancer and dementia, studies on this population are sparse.

Two previous reviews on cancer and dementia indicated that patients with cancer and dementia experience worse survival than those without dementia (6,11). The possible explanations, in addition to advanced age, included late stage at cancer diagnosis (12–16) and suboptimal cancer treatment options (6,11). In addition, there is prevailing uncertainty in determining appropriate treatment in the context of a patient with both cancer and dementia. This is compounded by the difficulty of balancing disease-specific guidelines with patient preferences, quality of life, and life expectancy of older adults with both cancer and dementia (17). While the benefits of certain treatments and shared decision-making for patients with either cancer (18–21) or dementia (22–24) are well documented, research that documents the impact of dementia on cancer treatment decision-making is scarce. In addition, there are no current guidelines on how to treat these patients.

Existing reviews tended to focus on treatment decision-making from the aspects of clinicians, while caregiver and patient perspectives are currently lacking. In their review, Hopkinson et al. (11) found no studies about patient and caregiver treatment preferences or any qualitative research on the experience of patients with dementia receiving cancer treatment (11). In addition, the authors excluded studies focusing on end of life/terminal care. McWilliams et al. (2018) included 47 studies of quantitative and qualitative methodologies to estimate the prevalence of cancer and dementia, describe cancer related experiences, including cancer screening, diagnosis, and treatment decisions, for patients, caregivers, and clinicians, and finally describe cancer-related outcomes for people with cancer and dementia (6). Similar to Hoskinson et al. (11), they found no studies that explored palliative or end-of-life decision-making (6,11).

Our review framework differs from previous ones in that it aimed to provide an integrated assessment that follows the care continuum for patients with cancer and dementia. We conducted a mixed studies review with the goal of assessing the current knowledge and gaps across three main concepts (Figure 1) related to the dual illness trajectory for cancer and dementia: 1) decision-making processes for cancer treatment from the perspective of patients, caregivers, and clinicians; 2) impact of a dementia diagnosis on curative and supportive treatment; and 3) all-cause and/or cancer-specific mortality. We included both quantitative and qualitative studies and summarized range of quantitative measures, such as odds ratios (ORs) and hazard ratios (HRs) whenever possible and performed narrative review otherwise.

**Figure 1.**
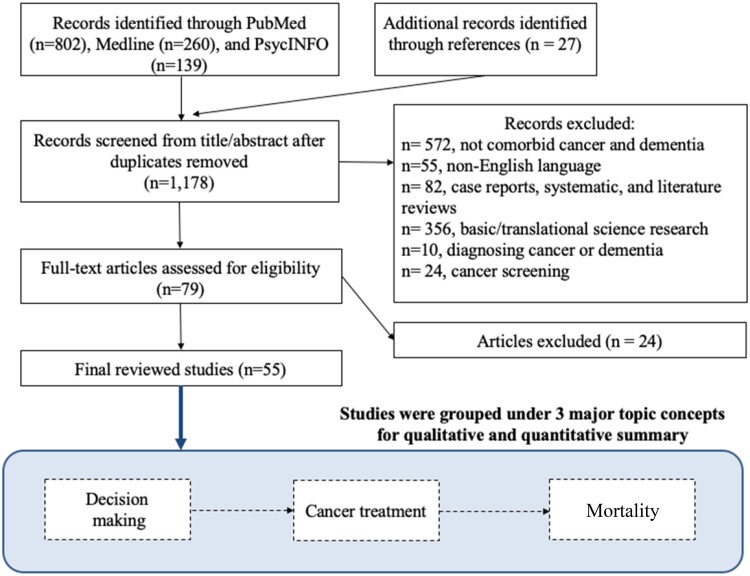
Study design and mixed studies review paper selection.

## Methods

We conducted a structured literature search in PubMed, Medline, and PsycINFO databases using the following terms: “cancer” AND “dementia” OR “Alzheimer’s” AND “treatment decision-making” OR “management” to identify English language peer-reviewed articles published between January 2004 and February 2020. We decided a priori to set the starting date as January 2004 to ensure the identification of the most clinically relevant research to date given that practices in treating cancer and dementia are continually evolving; this also reduces overlap between the present study and previous reviews. We followed the preferred reporting guidelines items for systematic reviews and meta-analyses (PRISMA) in screening relevant articles (Figure 1). We identified initially 1178 records, as well as 27 additional papers manually identified from reference lists of previous reviews (6,11). Three reviewers (YC, KD, BL) independently screened titles and abstracts for inclusion. To resolve any disagreements, the reviewers met to reach a consensus. If a consensus was not met, the opinion of the Cancer and Aging Working Group consisting of 8 members at the Institute for Translational Epidemiology was sought. We included articles that address any of the three topics outlined in Figure 1, and excluded articles that were case reports, studies on basic biomolecular mechanism of cancer and dementia, studies on cancer screening, and studies on cognitive impairment resulting from cancer treatment. Through this process, 55 studies were included in the current mixed studies review.

Three researchers (YC, KD, and BL) performed quality assessment of the included studies using the 2018 Mixed Methods Appraisal Tool (MMAT), which was chosen due to its flexibility to permit appraisal of studies with qualitative, quantitative, or mixed methods design, and its application in previous reviews of cancer and aging (6,25). Other researchers investigating the MMAT’s reliability have found that agreement between reviewers for the criteria was reasonable and reported that it was easy to use (26). The MMAT criteria include two screening questions and five items to appraise five study categories (qualitative, quantitative [randomized controlled trials], quantitative nonrandomized, quantitative descriptive, and mixed methods). We rated each item using a score of 0, 0.5, or 1 for studies that did not, somewhat, and fully meet the evaluation criteria, respectively. All studies received an overall quality score based on the appropriateness of study design, collection methods, completeness of data, risk of bias, and coherence. Scores ranged from 1 (low) to 5 (high) and no study was excluded on the basis of quality assessment.

We summarized the reported odds ratios or hazard ratios and their 95% confidence intervals (CIs) to assess the association of cancer treatment received (yes/no) and dementia status (with/without) among cancer patients. Although odds ratios and hazard ratios were the most commonly reported measures of association, a variety of other measures, such as relative risk (RR) and mortality risk ratio (MRR), were reported in a handful of studies. No curative treatment included the receipt of supportive treatment such as hospice or palliative care, while curative treatment included surgery, chemotherapy, and radiation. All-cause and cancer-specific mortality were summarized using hazard ratios, mortality risk ratios, and odds ratios, and their 95% confidence intervals for studies that compared cancer patients with and without dementia. We did not perform meta-summary of ORs and HRs, because the effect sizes were not similarly computed and there was no clear way to transform them to the same metric.

## Results

### Studies Overview

In Table 1 we grouped the identified 55 studies according to the three themes outlined in Figure 1: 1) cancer treatment decision-making (n = 22), (27–48) 2) cancer treatment types (n = 19) (39,49–64,66,74), and 3) mortality (n = 27) (33,49,50,52,55,57,60,62–81). Forty-eight studies reviewed were quantitative studies. The majority of studies were conducted in the US (n = 28), followed by the UK (n = 6), Japan (n = 6), France (n = 4), Netherlands (n = 3), Taiwan (n = 2), Australia (n = 2), and one each from Italy, Sweden, Denmark and Canada. Study designs included: cohort (n = 20), descriptive (n = 17), cross-sectional (n = 9), narrative (n = 4), mixed methods (n = 3), and case-control (n = 2).

**Table 1.** Summary of included studies, by three concepts: decision-making processes, cancer treatment, and mortality.

Concept category	Study	Study type	Data source (Country)	Sample size	Cancer type (stage)	Dementia ascertainment	Mixed methods appraisal tool (MMAT) score
Decision	Sherwood, et al., 2004 (44)	Cross-sectional	Two national brain tumor support groups and one internet support group for the bereaved (US)	43 caregivers (CGs)	Brain (I-III)	Not available	4.5
Decision	Kimmick, et al., 2014 (32)	Cross-sectional	National cancer registry (US)	6439 women	Breast (0-III)	Clinicians and outpatient facilities; Adult comorbidity evaluation Index (ACE-27)	5
Decision	Smyth, 2009 (45)	Narrative	Alzheimer's Disease research center registry (US)	21 CGs of women with dementia	Breast (not reported)	Not available	4.5
Decision	Morgan, et al., 2015 (37)	Mixed methods	Purposive sampling of health-care professionals (HCPs) from registry data across 14 sites; UK association of breast surgery (UK)	34 HCPs	Hypothetical breast (operable)	Hypothetical dementia not defined	4.5
Decision	Morgan, et al., 2017 (38)	Cross-sectional	Cross-sectional questionnaires (UK)	258 HCPs	Hypothetical breast (operable)	Hypothetical mild to severe cognitive impairment	5
Decision	Cook & McCarthy, 2018 (27)	Narrative	Large cancer care service at a public hospital (Australia)	9 HCPs	Hypothetical cancer	Hypothetical dementia	5
Decision	Rietjens, et al., 2005 (42)	Mixed methods	Members of the Panel of the “consumers’ association and professional registries (Netherlands)	1388 people from the general public; 391 clinicians	Hypothetical cancer (metastatic)	Hypothetical progressive dementia	5
Decision	Wong, et al., 2012 (48)	Cross-sectional	Australasian college for emergency medicine (Australia)	190 fellows, 176 trainees	Hypothetical cancer (metastatic)	Hypothetical dementia	4.5
Decision	Niemier, et al., 2018 (40)	Cross-sectional	All general practitioners (GPs) in Lorraine (France)	430 GPs	Hypothetical cancer (primary)	Hypothetical cognitive impairment	5
Decision	Dening, et al., 2016 (28)	Mixed methods	Four sites of memory clinics (UK)	60 dyads (a person with early dementia and preserved capacity and their family caregiver)	Hypothetical cancer (terminal)	International Statistical Classification of Diseases and Related Health Problems 10 (ICD10), Mini-Mental rtate examination (MMSE)	5
Decision	Mohile, et al., 2018 (36)	Cross-sectional	Two geriatric oncology studies (US)	305 community oncologists	Hypothetical pancreatic Cancer (metastatic)	Hypothetical dementia	5
Decision	Girones, 2005 (30)	Descriptive	Single center (US)	83 patients	Lung (I-IV)	Comprehensive geriatric assessment (CGA)	4.5
Decision	Ogawa, et al., 2010 (41)	Descriptive	Single hospital (Japan)	2000 patients	Mixed cancer types (I-IV, recurrence, unknown)	Consultation-liaison psychiatrists	4.5
Decision	Iritani, et al., 2011 (31)	Cohort, retrospective	Single hospital (Japan)	134 patients	Mixed cancer types (I-IV)	MMSE, Diagnostic and Statistical Manual of Mental Disorders, 4th edition (DMS-IV)	5
Decision	McWilliams, et al., 2018 (35)	Narrative	Regional cancer center (UK)	10 patients, 9 caregivers 12 HCPs	Mixed cancer types (I-IV)	Clinicians	5
Decision	Russo, et al., 2018 (43)	Descriptive	Comprehensive cancer centre Léon Bérard (France)	266 patients	Mixed cancer types (local, locally advanced, metastatic, missing)	Multidimensional geriatric assessment (MGA)	3.5
Decision	Flood, et al., 2006 (29)	Descriptive	Single hospital (US)	119 patients	Mixed cancer types (not reported)	ICD9	5
Decision	Witham, et al., 2018 (47)	Narrative	Psycho-oncology unit at a regional cancer center (UK)	7 CGs	Mixed cancer types (not reported)	GPs	4.5
Decision	Malik, et al., 2019 (34)	Descriptive	Academic geriatric oncology clinic (Canada)	82 patients	Mixed cancer types, genitourinary primary (not reported)	Mini-Cog; Rowland Universal Dementia Assessment Scale; MMSE	5
Decision	van der Poel, et al., 2014 (46)	Cross-sectional	Dutch-Belgian cooperative trial group for haemato-Oncology (HOVON) (Netherlands)	94 Hematologists	Non-Hodgkin’s lymphoma (NHL) (not reported)	Hypothetical dementia	5
Mortality	Ording, et al., 2013 (76)	Cohort, retrospective	National medical registries (Denmark)	47 904 patients and 237,938 matched controls	Breast (local, regional, distant, unknown)	ICD8/10, Charlson comorbidity index (CCI)	5
Mortality	Patnaik, et al., 2011 (78)	Descriptive	SEER (US)	64 034 patients and 37,306 controls	Breast (I-IV, unknown)	ICD9, CCI	5
Mortality	Louwman, et al., 2005 (72)	Cohort, prospective	Eindhoven cancer registry (Netherlands)	8966 patients	Breast (I-IV; unknown)	Not available	4.5
Mortality	Raji, et al., 2008 (79)	Cohort, retrospective	SEER (US)	106 061 patients	Breast, prostate, colon (I-IV; unknown)	ICD9	5
Mortality	Ganguli, et al., 2005 (69)	Cohort, prospective	Monongahela valley independent elders survey (US)	1670 adults	Cancer type not reported	DSM-III, National institute of neurological and communicative diseases and stroke/alzheimer's disease and related disorders association (NINCDS-ADRDA), Clinical dementia rating scale (CDRS)	5
Mortality	Neuman, et al., 2013 (75)	Cohort, prospective	SEER (US)	12 979 patients	Colon (I-III)	ICD9	5
Mortality	O'Rourke, et al., 2008 (77)	Cohort, retrospective	Portland veteran’s administration hospital (US)	160 patients	Esophageal (regional, advanced)	DSM-IV	5
Mortality	Mohammadi, et al., 2015 (73)	Cohort, retrospective	Patient register (Sweden)	7134 patients	Leukemia/myeloma (not reported)	ICD10	5
Mortality	Islam, et al., 2015 (71)	Cohort, retrospective	Nebraska cancer registry (NCR) and Nebraska hospital discharge data (NHDD) (US)	5683 patients	Lung (localized, regional, distant)	Not available	4.5
Mortality	Chang, et al., 2014 (65)	Cohort, retrospective	South London and Maudsley NHS foundation trust (SLAM) Biomedical research centre (BRC) case register; Thames cancer registry (TCR) (UK)	28 477 patients	Mixed cancer types (localized and advanced)	ICD10	4.5
Mortality	Rozzini and Trabucchi, 2009 (80)	Cohort, retrospective	Poliambulanza Hospital (Italy)	2843 patients	Mixed cancer types (metastatic)	DSM-IV	4
Mortality	Hirooka, et al., 2020 (70)	Descriptive	Randomly selected nursing agencies (Japan)	508 patients (surveys completed by home visiting nurses)	Mixed cancer types (not reported)	Medical records	4.5
Mortality	Zaorsky, et al., 2017 (81)	Descriptive	SEER (US)	1 895 788 patients	Mixed cancer types (not reported)	ICD9/10	4.5
Mortality	Chen, et al., 2015 (66)	Cohort, prospective	Longitudinal Health Insurance database 2005 (Taiwan)	37,411 patients	Mixed cancer types and stages	ICD9, prescription medication	5
Mortality	D’Amico, et al., 2010 (68)	Cohort, retrospective	Chicago prostate dancer Center (US)	6647 men	Prostate (multiple stages)	Alzheimers disease assessment scale (ADAS)	5
Mortality, decision	Lee, et al., 2018 (33)	Cohort, retrospective	National claims database (Taiwan)	37 289 patients	Cancer type not reported	ICD9	5
Mortality, treatment	Abe, et al., 2011 (49)	Descriptive	Hachioji medical center (Japan)	31 patients	Acute myeloid leukemia (AML) (de novo, AML/myelodysplastic syndrome [MDS])	MMSE, single photon emission computed tomography (SPECT)	5
Mortality, treatment	Shinden, et al., 2017 (62)	Descriptive	Single hospital (Japan)	773 patients	Breast (0-III)	Clinicians	4
Mortality, treatment	Baillargeon, et al., 2011 (50)	Cohort, retrospective	SEER (US)	80 670 patients	Colon (I-IV, unknown)	ICD9	5
Mortality, treatment	Chen, et al., 2017 (67)	Cohort, retrospective	SEER (US)	4,73 patients	Colon (III)	ICD9, Medication	5
Mortality, treatment	Robb, et al., 2010 (60)	Retrospective case-control	Geriatric oncology program at an NCI-designated comprehensive cancer Center (US)	258 patients	Mixed cancer types (0-IV)	MMSE	4
Mortality, treatment	Legler, et al., 2011 (57)	Cross-sectional	SEER (US)	27 166 hospice users	Mixed cancer types (advanced)	ICD9	5
Mortality, treatment	Galvin, et al., 2018 (52)	Descriptive	Four cancer registries and three prospective cohort studies from Gironde (France)	450 patients	Mixed cancer types (yes/no/unknown advanced stage)	DSM-IV, Clinicians	5
Mortality, treatment	Bradley, et al., 2008 (64)	Descriptive	Medicaid and medicare data merged with Michigan tumor registry (US)	1907 patients	Mixed cancer types and stages	ICD9	5
Mortality, treatment	Kedia, et al., 2017 (55)	Cohort, retrospective	Centers for medicare and medicaid Services (CMS) (US)	96 124 patients	Mixed cancer types excluding nonmelanoma skin cancer (not reported)	ICD9	5
Mortality, treatment	Neuman, et al., 2013 (74)	Descriptive	SEER (US)	31 574 patients	Primary colon adenocarcinoma (local, regional, unstaged)	ICD9	5
Mortality, treatment	Wongrakpanich, et al., 2017 (63)	Descriptive	Single medical center (US)	3460 patients	Solid tumors (0-IV)	DSM-IV	4.5
Treatment	Gorin, et al., 2005 (53)	Cohort, retrospective	SEER (US)	5460 patients	Breast (in situ, I-III)	ICD9	5
Treatment	Fleming, et al., 2014 (51)	Descriptive	Four state cancer registries (US)	3116 patients	Colon (I-III), Rectal (III)	ACE-27	4.5
Treatment	Gupta & Lamont, 2004 (54)	Cohort, retrospective	SEER (US)	17 507 patients	Colon (I-IV)	ICD9	5
Treatment	Saffore, et al., 2018 (61)	Cohort, retrospective	SEER (US)	10 626 patients	Diffuse large B-cell lymphoma (I-IV)	ICD9/10	5
Treatment	Kodama, et al., 2009 (56)	Descriptive	Clinics specialized in home care from 10 different locations in eight localities (Japan)	15 patients	Hematologic	Not available	4.5
Treatment	Monroe, et al., 2012 (58)	Cross-sectional	Nursing Homes (US)	48 patients	Mixed cancer types (advanced)	ICD9	5
Treatment	Monroe, et al., 2013 (59)	Descriptive	Nursing Homes (US)	55 patients	Mixed cancer types (terminal)	ICD9	5
Treatment, decision	Morin, et al., 2016 (39)	Case-control	National hospital registry (France)	26 782 patients	Mixed cancer types and stages	ICD10	5

The sample size of studies ranged from 7 to 1 895 788 participants with study populations derived from the Surveillance, Epidemiology, and End Results (SEER; n = 11), other national registries (n = 18), single medical centers (n = 19), multiple medical centers (n = 2) nursing homes (n = 3), support groups (n = 1), and a cooperative trial group. The studied cancer types varied widely with the majority including populations with either breast (n = 10), colon (n = 7), hematologic (n = 5), or some combination of cancers (n = 33). Eight studies used hypothetical cancer scenarios, of which four described scenarios of metastatic or terminal cancer.

The MMAT scores of the included studies ranged from 3.5 to 5 out of a possible 5 (Table 1), and the overall average was 4.75 (standard deviation = 0.35). Reasons for low scores included non-representativeness of the study samples to the target population, failure to disclose response rate, unclear definitions in outcome or exposure variables, inadequate adjustment for confounders in the design and/or analysis, or insufficient description of qualitative data collection methods used. Among all studies, excluding those that used hypothetical scenarios, 5 studies did not specify how dementia ascertainment was conducted, while inconsistent definitions of dementia were used in the remaining ones (Table 1). As shown in Table 1 and Supplementary Tables 1 and 2 (available online), the specific codes used to identify dementia varied widely among studies, even among those that used the International Classification of Diseases.

### Cancer Treatment Decision-Making

Twenty-two out of the 55 studies we reviewed investigated the impact of dementia on cancer treatment decision-making and disease management, with most of them being quantitative studies (Table 2). Using a narrative review, we summarized the common and unique aspects faced by patients, caregivers/family members or relatives, and clinicians.

**Table 2.** Impact of dementia on cancer treatment decision-making from the perspectives of patient/caregiver/family and clinicians.

Study	Perspective
Patient/Caregiver/Family Perspective	
Niemier, et al., 2018 (40)^a^	Patient’s wishes were not always in line with those of his/her family or doctor. An evaluation of quality of life is often subjective and difficult to achieve.
Witham, et al., 2018 (47)	Communication is difficult for caregivers as their roles were minimized by patients and health-care professionals.
	The decision-making process of caregivers was inspired by realism in terms of both quality of life and prognosis.
	Since patients usually cannot answer for themselves, key contacts (caregivers) were needed.
	Caregivers were concerned with maintaining personal integrity of patients.
	Some caregivers/relatives have power of attorney for their relative with dementia, which makes their involvement in decision-making a legal issue.
McWilliams, et al., 2018 (35)	Patients are not as actively engaged; families voiced patients’ opinions for them.
	Relatives assumed the role of proxy health-care professionals (not always formalized).
	Caregivers ensure timely access to cancer specialists and collate information about dementia before the first visit.
	Caregivers needed more time for communication (it was all “too fast”).
	Sometimes caregivers were not clear on the proposed treatment and associated risks.
	Written information about treatments was not always useful; caregivers may not have time to go over it or it was confusing because it was not dementia-oriented.
	Most of the time, families saw that dementia had a direct impact on cancer treatment decision-making, whereas other times it was not as clear.
Morin, et al., 2016 (39)	Older adults with dementia have been found to be more often oriented toward the relief of discomfort and quality of life than toward longer survival.
	Patients are less likely to request an aggressive approach.
	Impaired patient-clinician communication may facilitate earlier discontinuation of anticancer therapy.
	Diagnosis of dementia could lead relatives to give higher priority to quality of life over life expectancy, to recognize the palliative nature of the situation, and reconsider the benefit of anticancer treatments.
Dening, et al., 2016 (28)	In an advanced cancer scenario, patients expressed lower preferences for all treatments (antibiotics 47%; Cardiopulmonary resuscitation (CPR 30%; tube feeding 37%). Caregivers had similar views to patients with dementia overall (antibiotics, 56%; CPR, 32%; tube feeding, 37%).
	In an advanced cancer scenario, the agreement between patients and caregivers was low (antibiotics, 24%; CPR, 27%; tube feeding, 39%).
	Both patients and caregivers showed uncertainty about their preferences for end of life treatment choices.
	Caregivers often find making health-related decisions for the patient they care for stressful, especially those concerning end-of-life care.
Clinician Perspective	
Mohile, et al., 2018 (36)^a^	Community oncologists incorporated patient age, functional impairment, and cognitive impairment into decision-making for treatment.
	≤25% of community oncologists rated themselves as “very confident” in assessing and intervening for function, falls, and dementia.
	Clinician beliefs/confidence in management of age-related health issues did not influence chemotherapy decisions.
	Many oncologists believed that geriatric training is essential for the care of older cancer patients and would appreciate additional training in age-related topics.
Niemier, et al., 2018 (40)^a^	Patient’s wishes, quality of life, and comorbidities were the three criteria most frequently expressed to be the most important by general practitioners in 2014; these criteria were emphasized less in 2015.
	Cancer management is limited especially in cases of cognitive impairment since general practitioners may rely on their own perception of the patient.
Witham, et al., 2018 (47)	Health-care professionals within oncology need to create more adaptable treatment pathways that are more responsive to those with cognitive impairment.
	Stigma associated with dementia needs to be re-examined.
	Health-care professionals need to reflect on communicating effectively with caregivers.
McWilliams, et al., 2018 (35)	Health-care professionals relied on informal caregivers to identify and manage treatment side effects.
	Using information from caregivers, clinicians can assess capacity to consent, prepare for appropriate communication, have insight about cognitive abilities, and involve dementia-specific support from the beginning.
	Clinicians acknowledged that extra time is needed to communicate with this group.
	Health-care professionals had limited awareness of dementia’s impact on cancer diagnostic investigations.
	Individual impact of dementia should ideally be known at initial multidisciplinary team meetings.
	When appropriate adjustments to care were not made, some health-care professionals were aware that they could have intervened earlier.
Lee, et al., 2018 (33)	Most clinicians and family members chose palliative care for older cancer patients diagnosed with dementia because of the discomfort caused by cancer treatments.
Russo, et al., 2018 (43)	The Multidimensional Geriatric Assessment (MGA) revealed malnutrition (47%), cognitive/mood impairment (48%), functional decline (53%), and led to adjust medical care through reinforcing health status and fostering successful completion of cancer treatment plan for 259 (97%) patients.
	The MGA changed cancer treatment in 47 (18%) patients.
Morgan, et al., 2017 (38)^a^	Health-care professionals were less likely to prefer surgery and more likely to opt for primary endocrine therapy for patients with moderate and severe cognitive impairment: (1) for surgery vs equal preference, relative risk ratio (RRR= 0.32 (0.24 to 0.42) among patients with moderate impairment and RRR = 0.01 (0.01 to 0.03) among patients with severe impairment; (2) for primary endocrine therapy (PET) vs equal preference, RRR = 3.67 (2.01 to 6.48) among patients with moderate impairment and RRR = 21.45 (7.01 to 65.57) among patients with severe impairment
	Vague and conflicting guidelines: national guidelines recommend patients with operable breast cancer be treated with surgery “irrespective of age” while the International Society of Geriatric Oncology (SIOG) and the European Society of Breast Cancer Specialists (EUSOMA) recommend PET be offered to patients with short life expectancy (<2-3 years), are unfit, or refuse surgery.
	No specific guidelines for patients with dementia exist.
Morin, et al., 2016 (39)	Clinicians might be more reluctant to prescribe aggressive treatments to patients with cancer and dementia near the end of life.
	Inability of patients to provide consent may prompt withholding of curative treatment.
	Practical difficulties affect clinical investigations (imaging, biopsy, colonoscopy, etc.), provision of intravenous (IV therapy, radiation therapy, and blood transfusions.
	There is a need for qualitative studies to gain better understanding of the decision-making process leading to limitation or discontinuation of cancer treatments in individuals with dementia
Girones, 2015 (30)	Regarding the informing of a cancer diagnosis, patient attitudes differed from clinician attitudes. Characteristics that clinicians considered important enough to not inform the patient of cancer (age, dementia, depression, frailty) were correlated to a stronger desire to be informed on the patient’s part.
Morgan, et al., 2015 (37)^a^	Clinician opinions differ on the best way to treat women >70 years with operable breast cancer, especially if they have dementia (PET vs surgery).
	89% of clinicians rated dementia as very important or important in making cancer treatment decisions.
	41.1% agreed that “all women ≥70 years with operable estrogen receptor positive (ER+) breast cancer, who had significant dementia should be treated with PET.”
	No guidelines were available for this population.
	Patient’s inability to provide informed consent complicates cancer treatment decision-making.
Kimmick, et al., 2014 (32)	Dementia (OR = 0.45 [0.24 to 0.82]) predicted lack of guideline concordance, which was modeled on tumor size, node status, and hormone receptor status.
van der Poel, 2014 (46)^a^	Dementia was included in treatment decision-making in older patients with a hematological malignancy: 73% responded always included; 17% responded often included; 10% responded sometimes included.
Wong, et al., 2012 (48)^a^	For a hypothetical older nursing-home resident with dementia, metastatic cancer and possible septic shock, 10.7% (39/366) of emergency clinicians chose commencing full treatment, changing little (21/365, 5.8%) with a directive requesting full treatment.
	The patient's presentation and history (189/375, 50.4%) had more impact than legal obligations (14/375, 3.7%) in influencing the decision.
Ogawa, et al., 2010 (41)	General support and psychological interventions are needed for patients with cancer and dementia.
	Planning for and delivery of home care services is often fragmented.
Flood, et al., 2006 (29)	Recognition of cognitive or functional disability in older patients with cancer was often missed using standard oncology performance assessment scales.
	Management of geriatric syndromes has a direct influence on cancer treatment.
Rietjens, et al., 2005 (42)^a^	When presented with a vignette of a patient with metastasized cancer and progressive dementia, acceptance of active ending of life at the request of a terminally ill patient was 36% among surveyed Dutch clinicians.
Malik, et al., 2019 (34)	Final oncological treatment plans were influenced by the geriatric oncology clinic's recommendations in 18 (60.0%) of the 30 patients with screen-detected cognitive impairment. Eleven (36.7%) out of 30 cases had an unchanged final treatment plan after the comprehensive geriatric assessment (CGA), 10 (33.3%) had reduced treatment intensity and 7 (23.3%) involved a change in treatment to best supportive care.
	Among the 17 patients with a reduction in treatment intensity or change to best supportive care, cognitive impairment was a factor in 7 (70.0%) of the 10 cases with reduced treatment intensity and in 5 (71.4%) of the 7 cases with change to best supportive care.
	A standardized protocol for downstream workup of cognitive impairment should be considered for a more uniform diagnostic and management approach.
Cook & McCarthy, 2018 (27)^a^	Two themes that underlie the complicated processes of risk-benefit assessment in treatment decision-making: the unequal distribution of capital and power between health workers; and whether older adults with cancer and dementia are assessed as individuals or embedded in supportive social networks (individual versus relational autonomy).
	Cancer treatment might not be in the best interests of an older adult with dementia.
	Heavy burden on clinicians to reach a sound decision, give advice to patients and their families/caregivers, and to provide the best outcome for the older adult.
	Focus on the individual and pathology is more likely to marginalize the opinion of the older adult with cognitive impairment due to the power of medical and social beliefs on dementia, and the social position of the clinician with their high levels of capital.
Hirooka, et al., 2020 (70)	Medical care professions should support the decision-making process and engage in end-of-life discussions to achieve a good death, especially for patients with cancer and dementia.
	Since some patients with moderate dementia can participate in decision-making through shared decision-making, medical professionals should make efforts to develop shared decision-making strategies.

a Dementia ascertainment unavailable or not applicable.

***Perspectives of the patient and their caregiver(s).*** As shown in Table 2, a majority of caregiver/family members and clinicians tended to choose less aggressive treatment, consistent with previous reviews (6,11), and were more often oriented toward the relief of discomfort, and gave higher priority to quality of life over life expectancy (28,33,37–39,44,47). When considering treatment options, caregivers were also concerned with maintaining the personal integrity and quality of life for patients (47). Caregivers of patients with primary malignant brain tumors (PMBTs) and Alzheimer’s disease who received hospice care regarded the services as “invaluable” and “empathetic,” especially because it allowed them to keep the care-recipients at home until death (44). On the other hand, in a sample of 134 cancer patients with and without dementia, Iritani et al., showed that 8% of patients with dementia sought medical consultation compared to 63% of patients without dementia (31).

The importance of caregivers and family members in the treatment decision-making process was evident in most studies reviewed (28,33,35,39,44,47). They often acted as the patient’s voice and support system, relaying health information between patients and clinicians. For example, caregivers collected health-care information related to the patient’s dementia before their initial visits with oncologists (35) (Table 2), requested information related to treatment options (35,44,47), and communicated with clinicians when patients had difficulties doing so themselves. However, caregivers also encountered difficulties in communicating with clinicians (35,44,47), making health-related decisions, especially in regard to end-of-life care (47) and understanding the impact of dementia on their own treatment decision-making (35). In addition, caregivers/family found it was difficult to obtain support and guidance to manage patient symptoms at home, and had difficulty in obtaining hospice services (44).

***Perspective of Clinicians.*** Dementia in patients influenced most clinicians to reconsider what type of cancer treatment to offer. Four studies revealed that clinicians were less likely to opt for aggressive therapy for several reasons: inability of patients to provide informed consent (37,39), practical difficulties that arise from having dementia (39), high level of patient discomfort (33), vague and conflicting guidelines or lack of guidelines (37,38), and clinicians’ personal beliefs (37). A survey on treatment options for operable breast cancer showed that severe dementia among patients had a great impact on treatment recommendations by health care professionals, with greater preference given to primary endocrine therapy (PET) over surgery (37). Though some surgeons preferred less aggressive therapy, others were more inclined to provide aggressive treatment. Clinical tools such as the Multidimensional Geriatric Assessment (MGA) and the Mini Mental Status Examination (MMSE) that assess for cognitive impairment are used to facilitate decision-making (43). However, inconsistent use of these tools and inconsistent guidelines represent a barrier for this growing population.

***Integrated perspectives.*** The involvement of these two parties (patients/caregivers/family members and clinicians) in the decision-making process varied. Patients with both cancer and dementia tended to be less involved in their disease management and cancer treatment decision-making (28,35), while often relying on caregivers and family members to navigate decision-making and treatment information (35,39,44). However, misalignments in treatment preferences between patients and their caregivers may exist. Dening et al. (28) performed a cross-sectional narrative study interviewing 60 dyads of patients with early dementia and their caregivers. They found that in hypothetical scenarios of advanced cancer, agreement about treatment preferences (antibiotics, CPR, tube feeding) between patients with dementia and caregivers was low (28). In addition, patients’ preferred treatment options are not always known by clinicians (35,47), one reason being the limited capacity of patients with dementia to communicate their feelings or perspectives. Two studies, one conducted in a nursing home and the other in a hospital, reported that patients with dementia exhibit few signs of discomfort and complain less frequently of pains associated with cancer than those without dementia (31,58). Patients with breast cancer and dementia were also less likely to be aware of their own cancer (62). As a result, managing the pain associated with having both conditions is difficult. Two studies found low opioid administration for this patient population (58,59), and one found that for multiple cancer types (stages I-IV) patients with cancer and dementia received less pain medication than patients without dementia (31).

While clinicians tend to prescribe less aggressive treatment, which aligned with patient and caregiver/family preferences overall, clinicians also face several challenges, including lack of consistent guidelines in treatment decision making from professional organizations (32,37,38). Different opinions among health care professionals exist when it comes to how to treat patients with both dementia and cancer (37,38,42,48). This may be due to clinicians’ difficulties in accurately evaluating a patient’s quality of life (40). One study hypothesized that impaired communication between the patient and clinician may enable early discontinuation of curative cancer treatment (39). They suggest that dementia likely interferes with the “mutually reinforcing process…[of] not giving up” between patients and clinicians (39). As a result, there is also interest in assessing the confidence among clinicians when treating patients with cancer and dementia. In a survey of community oncologists who treat patients with pancreatic cancer, 25% or less rated themselves as “very confident” in assessing for and intervening with dementia (36). While this study found that clinician beliefs or confidence did not influence their decisions for offering chemotherapy, they emphasized the need for additional geriatric training for oncologists in age-related topics such as dementia. Overall, clinicians’ treatment decision-making processes varied widely due to differences in personal opinion, lack of specific guidelines, difficulty in obtaining informed consent, and expectations of patient discomfort.

### Cancer Treatment among Patients with Cancer and Dementia

In general, patients with both cancer and dementia tended to receive less cancer-related treatment, including chemotherapy, surgery, or radiation (Table 3); and were more likely to receive no treatment, including hospice and palliative care than patients with cancer and without dementia (Table 4).

**Table 3.** Reported measures of receiving curative cancer treatment among cancer patients with vs without dementia.

Study	Cancer type, stage	Treatment type (Yes vs. No)	Measures of association (95% CI), *P* value	Adjusted measures of association (95% CI), *P* value
Bradley, et al., 2008 (64)	Breast, Lung, colorectal, or prostate (local and regional)	Cancer-directed surgery	—	aOR = 1.3 (0.71 to 2.4)^a^, *p* = .39
Gorin, et al., 2005 (53)	Breast, I-III	Mastectomy^b^	OR= 0.68 (0.60 to 0.76), *p* < .001	—
Gorin, et al., 2005 (53)	Breast, I-III	Surgery	OR= 0.30 (0.24 to 0.38), *p* < .001	aOR= 0.60 (0.46 to 0.81)^c^
Gupta & Lamont, 2004 (54)	Colon, I-III	Surgical resection	—	aOR= 0.43 (0.33 to 0.70)^d^
Gupta & Lamont, 2004 (54)	Colon, III	Adjuvant 5-fluorouracil (5FU; among patients with surgical resection)	—	aOR= 0.21 (0.13 to 0.36)^e^
Gorin, et al., 2005 (53)	Breast, I-III	Chemotherapy	OR= 0.30 (0.23 to 0.38), *p* < .001	aOR= 0.44 (0.34 to 0.58)^c^
Fleming, et al., 2014 (51)	Colon, III	Chemotherapy	—	aOR = 0.11 (0.013 to 0.90)
Morin, et al., 2016 (39)	Mixed cancer types and stages	Chemotherapy in the last month of life	OR = 0.33 (0.31 to 0.36)	aOR = 0.33 (0.31 to 0.36)
Saffore, et al., 2018 (61)	Diffuse large B-cell lymphoma, I-IV	Any chemoimmunotheraphy	OR= 0.33 (0.26 to 0.40), *p* < .001	aOR = 0.44 (0.35 to 0.55)^f^, *p* < .001
Saffore, et al., 2018 (61)	Diffuse large B-cell lymphoma, I-IV	Rituximab and any chemotherapy	OR= 0.33 (0.26 to 0.40), *p* < .001	aOR = 0.44 (0.35 to 0.55)^f^, *p* < .001
Saffore, et al., 2018 (61)	Diffuse large B-cell lymphoma, I-IV	Anthracycline with or without other chemotherapy	OR= 0.33 (0.26 to 0.41), *p* < .001	aOR = 0.44 (0.35 to 0.55)^f^, *p* < .001
Galvin, et al., 2018 (52)	Mixed cancer types, yes/no/unknown advanced	Treatment administration	—	HR = 0.68 (0.47 to 0.99)^g^, *p* = .046
Gorin, et al., 2005 (53)	Breast, I-III	Radiation	OR = 0.24 (0.21 to 0.27), *p* < .001	aOR= 0.31 (0.23 to 0.41) for those who received BCS
Morin, et al., 2016 (39)	Mixed cancer types and stages	Radiation in the last month of life	OR = 0.49 (0.43 to 0.56)	aOR= 0.56 (0.49 to 0.65)^h^

a Adjusted for age, sex, insurance, comorbidity, cancer site and stage, and census-tract median income. — = Value not reported; CI = Confidence Interval; aOR = Adjusted odds ratio; OR = Odds ratio; HR = Hazard ratio

b Mastectomy vs breast-conserving surgery.

c Adjusted for age, sex, race, comorbidity, cancer presentation (nodal status, tumor size, and estrogen receptor status), and census-tract level poverty.

d Adjusted for age, sex, marital status, race, comorbidity, census-tract level poverty, urbanicity, and geographic region.

e Adjusted for age, sex, marital status, race, comorbidity, census-tract level poverty, urbanicity, geographic region, and histological grade.

f Adjusted for race, age, sex, Ann Arbor stage, and comorbidities at diagnosis.

g Adjusted for age, sex and stage at diagnosis.

h Adjusted for age, sex, race, comorbidity, cancer type, metastatic stage, year of death, type of hospital where death occurred.

**Table 4.** Reported measures of receiving no curative cancer treatment among cancer patients with vs without dementia.

Study	Cancer type, stage	Not receiving treatment (Yes vs. No)	Measures of association (95% CI), *P* value	Adjusted measures of association (95% CI), *P* value
Baillargeon, et al., 2011 (50)	Colon, all stages	No treatment	RR= 4.0 (3.6 to 4.6)	aRR= 2.5 (2.1 to 2.9)^a^
Baillargeon, et al., 2011 (50)	Colon, III	No chemotherapy	RR = 4.4 (3.7 to 5.3)	aRR = 3.2 (2.7 to 3.9)^a^
Bradley, et al., 2008 (64)	Breast, lung, colorectal, or prostate, local and regional	Hospice use	—	aOR = 1.0 (0.77 to 1.4), *p* = .85; aRR = 1.0 (0.84 to 1.2)^b^, *p* = .86
Gorin, et al., 2005 (53)^c^	Breast, I-III	No treatment	OR = 1.7 (1.7 to 1.8)	aOR = 1.5 (1.3 to 1.6)^d^
Legler, et al., 2011 (57)	Mixed cancer types, advanced	Hospice disenrollment	—	aOR = 1.2 (1.05 to 1.3)^e^, *p* < .01
Morin, et al., 2016 (39)	Mixed cancer types and stages	9 end of life treatment types	range OR = 0.46 to 0.98	range aOR = 0.40 to 0.97^f^
Neuman, et al., 2013 (74)	Colon, localized, regional, unstaged	No colectomy	—	aOR = 2.2 (1.8 to 2.7), *p* < .005
Wongrakpanich, et al., 2017 (63)	Solid tumors, 0-IV	Radiofrequency ablation	HR = 0.50 (0.27 to 0.94), *p* = .030	—

a Adjusted for age, sex, race and ethnicity, marital status, comorbidity, year of diagnosis, cancer stage, geographic region, and census-tract income measure.

—Value not reported; aRR = Adjusted risk ratio; aOR = Adjusted odds ratio; CI = Confidence Interval; RR = Risk ratio; OR = Odds ratio.

b Adjusted for age, sex, insurance, comorbidity, and census-tract median income.

c The original study compared the receipt of any treatment (Yes vs No) among cancer patients with vs without dementia. We used the reciprocal of what was reported so that the reference groups were consistent among studies in this table.

d Adjusted for age, sex, race, comorbidity, cancer presentation (nodal status, tumor size, and estrogen receptor status), and census-tract level poverty.

e Generalized estimating equations models with a gamma distribution and log link accounting for patient age, race, gender, marital status, site of primary cancer, days from hospice enrollment to death and region.

f Adjusted for age, sex, race, comorbidity, cancer type, metastatic stage, year of death, type of hospital where death occurred.

All but one of 4 analyses in the 3 studies that reported measures for the use of surgery found that patients with cancer and dementia were less likely to undergo surgery than patients with cancer and without dementia (OR range = 0.30-1.3, n = 4). Discrepancies existed regarding specific surgery types among patients who received surgeries. When comparing detailed surgery type for breast cancer, Gorin et al. 2005 reported that patients with Alzheimer’s disease received more breast-conserving surgery (38% vs 29%) and less mastectomy (62% vs 71%) than those without Alzheimer’s disease (53). An opposite finding was reported by Shinden et al. 2017, where patients with dementia underwent less breast conserving surgery (25% vs 41%) and more mastectomy (75% vs 59%) than those without dementia (62). Moreover, Bradley (64) reported that patients with Alzheimer’s disease received more cancer-directed surgery (OR = 1.3) than patients without Alzheimer’s. Women with breast cancer were also more likely to receive cancer-directed surgery than patients with colorectal, lung, or prostate cancer in this study.

There were 8 analyses overall in 6 studies that reported measures on the receipt of cancer-directed chemotherapy, hormone therapy, or chemo-immunotherapy among patients with cancer and dementia compared to patients with cancer and without dementia (Table 3). All studies found that patients with cancer and dementia received cancer-directed therapies less often than patients without dementia (OR and HR range = 0.11-0.68, n = 8).

Two studies that reported measures on the receipt of radiation found that patients with cancer and dementia received less radiation compared to patients without dementia (OR range = 0.24-0.56, n = 2, Table 3).

Lastly, there were 8 analyses in 7 studies that reported measures on the receipt of no curative cancer treatment, which included hospice and palliative care, among patients with cancer and dementia compared to patients with cancer and without dementia (OR, HR, and RR range = 0.40- 4.4, n = 8; Table 4). All but three studies found that patients with cancer and dementia received more hospice or palliative care than patients without dementia (Table 4) (39,63,64). In addition, levels of hospice service utilization were much higher in older Medicare beneficiaries with coexisting dementia and cancer diagnoses (21%) than those with a dementia-only diagnosis (12%), cancer-only diagnosis (5.5%), and neither dementia nor cancer (0.88%) (55). Of note, a study of patients with advanced cancer in the SEER database found that those with comorbid dementia dis-enrolled from hospice more often than those without comorbid dementia (12% vs 10%) (57).

### Mortality among Patients with Cancer and Dementia

A diagnosis of dementia was associated with higher mortality (HR and OR range = 0.92-5.8, n = 33) in patients with cancer and was associated with a shorter survival time (Table 5), consistent with what was found in previous reviews. 6,11 We found 31 of 33 analyses from 21 studies report that the cancer and dementia population had worse all-cause mortality (n = 25) and cancer-specific mortality (n = 6) compared to patients with either cancer or dementia only (Table 5).

**Table 5.** Reported measures of all-cause and cancer-specific mortality among cancer patients with vs without dementia.

Study	Cancer type (Stage)	Total no. of participants	Mortality outcome	Measure of association (95% CI)	Adjusted measure of association (95% CI)
Baillargeon, et al., 2011 (50)	Colon (I-IV)	80 670	Cancer-specific mortality (HR)	1.8 (1.7 to 1.8)	1.5 (1.4 to 1.6)^a^
Baillargeon, et al., 2011 (50)	Colon (I-IV)	80 670	All-cause mortality (HR)	2.0 (1.9 to 2.1)	1.6 (1.6 to 1.7)^a^
Bradley, et al., 2008 (64)	Mixed cancer types and stages	1907	Death within 3 months following diagnosis (OR)	1.3 (1.0 to 1.7)	—
Chang, et al., 2014 (65)	Mixed cancer types (localized and advanced)	28 477	General mortality (RR)	—	1.4 (1.2 to 1.6)^b^
Chen, et al., 2015 (66)	Mixed cancer types and stages	37 411	Mortality (HR)	5 (2.8 to 9.1)	—
Chen, et al., 2017 (67)	Colon (III)	4,573	Cancer-specific mortality (HR)	1.5 (1.3 to 1.6)	—
Galvin, et al., 2018 (52)	Mixed cancer types (yes/no/unknown advanced stage)	450	All-cause mortality among untreated cancer patients (HR)	2.8 (1.3 to 6.2)	—
Islam, et al., 2015 (71)^c^	Lung (localized)	5683	All-cause mortality (HR)	—	1.21 (0.59 to 2.5)^d^
Islam, et al., 2015 (71)^c^	Lung (regional)	5683	All-cause mortality (HR)	—	2.3 (1.2 to 4.5)^d^
Islam, et al., 2015 (71)^c^	Lung (distant)	5683	Al-cause mortality (HR)	—	1.1 (0.79 to 1.7)^d^
Kedia, et al., 2017 (55)	Mixed cancer types excluding nonmelanoma skin cancer (not reported)	96 124	Death (percentage)	28% (cancer/dementia) vs 9% (cancer only) vs 2% (no cancer/no dementia)	—
Kodama, et al., 2009 (56)	Hematologic	15	Death (percentage)	83% (dementia) vs 11% (no dementia)	—
Lee, et al., 2018 (33)	Cancer type not reported	37 289	Mortality rate (HR)	1.8 (1.7 to 1.9)	1.7 (1.6 to 1.8)^e^
Legler, et al., 2011 (57)	Mixed cancer types (advanced)	27 166	Hospital death (OR)	—	0.92 (0.70 to 1.2)^f^
Louwman, et al., 2005 (72)^c^	Breast (I-IV; unknown)	8966	All-cause mortality (HR)	2.3 (1.6 to 3.5)	(1.6 to 3.5)
Mohammadi, et al., 2015 (73)	Acute myeloid leukemia (not reported)	7134	Mortality rate ratio	1.5 (0.97 to 2.3)	(0.97 to 2.3)
Mohammadi, et al., 2015 (73)	Chronic myeloid leukemia (not reported)	7134	Mortality rate ratio	2.6 (1.3 to 5.3)	(1.3 to 5.3)
Mohammadi, et al., 2015 (73)	Myeloma (not reported)	7134	Mortality rate ratio	1.6 (1.2 to 2.2)	(1.2 to 2.2)
Neuman, et al., 2013 (75)	Colon (I-III)	12 979	1-Year Mortality (OR)	—	5.8 (3.1 to 11)^g^
Neuman, et al., 2013 (75)	Colon (I-III)	12 979	90-day Mortality (OR)	—	4.5 (2.4 to 8.5)^g^
Neuman, et al., 2013 (74)	Colon (localized, regional, unstaged)	31 574	Cancer specific mortality (HR)	2.1 (1.7 to 2.5)	—
Neuman, et al., 2013 (74)	Colon (localized, regional, unstaged)	31 574	All-cause mortality (HR)	1.9 (1.6 to 2.2)	—
O'Rourke, et al., 2008 (77)	Esophageal (regional, advanced)	160	Mortality (HR)	—	3.0 (1.4 to 6.6)^h^
Ording, et al., 2013 (76)	Breast (local, regional, distant, unknown)	285 842	Mortality rate ratio first year after cancer diagnosis	—	5 (3.6 to 6.8)^i^
Patnaik, et al., 2011 (78)	Breast (I-IV)	101 340	All-cause mortality (HR)	5.7 (5.3 to 6.1)	—
Raji, et al., 2008 (79)	Colon (I-IV; unknown)	106 061	All-cause mortality (HR)	—	1.7 (1.7 to 1.8)^j^
Raji, et al., 2008 (79)	Colon (I-IV; unknown)	106 061	Cancer specific mortality (HR)	—	1.5 (1.4 to 1.7)^j^
Raji, et al., 2008 (79)	Breast (I-IV; unknown)	106 061	All-cause mortality (HR)	—	2.2 (2.0 to 2.3)^j^
Raji, et al., 2008 (79)	Prostate (I-IV; unknown)	106 061	All-cause mortality (HR)	—	2.0 (1.9 to 2.1)^j^
Raji, et al., 2008 (79)	Breast (I-IV; unknown)	106 061	Cancer specific mortality (HR)	—	1.8 (1.6 to 2.0)^j^
Raji, et al., 2008 (79)	Prostate (I-IV; unknown)	106 061	Cancer specific mortality (HR)	—	1.7 (1.5 to 1.8)^j^
Shinden, et al., 2017 (62)	Breast (0-III)	773	Cancer death	0 (0%) vs 51 (7%) control	—
Wongrakpanich, et al., 2017 (63)	Solid tumors (0-IV)	3460	All-cause mortality (HR)	1.6 (1.3–2.1)	—

a Adjusted for age, sex, race and ethnicity, marital status, year of diagnosis, geographic region, and census-tract income measure. — = Value not reported; CI = confidence interval.

b Adjusted for age and gender.

c Dementia ascertainment unavailable or not applicable.

d Adjusted for age, race, sex, and histologic type.

e Adjusted for age, sex, cancer, stroke, chronic renal failure, liver cirrhosis, pressure injury, hospitalizations, receiving emergency services, nasogastric tube placement, oxygen supply, receiving CPR, and receiving endotracheal intubations.

f Adjusted for patient demographic (age at death, race, gender, marital status, and region) and clinical characteristics (site of primary cancer and log-transformed number of days from hospice enrollment to death.

g Adjusted for SEER registry, urban/rural residence, census track income and proportion of non-high school graduates, and year of diagnosis.

h Controlling for age and histology/cancer type.

i Adjusted for stage.

j Adjusted for age, ethnicity, sex (for colon cancer), marital status, Surveillance Epidemiology and End Results region, educational level.

In two studies, mortality by cancer stage between patients with and without dementia were compared. Islam et al. (2015) found that the all-cause mortality for lung cancer patients with vs without dementia was statistically significant among those with regional stage cancer (HR = 2.3 [95% CI = 1.2 to 4.5]), but not statistically significant for localized (HR = 1.2 [95% CI = 0.59 to 2.5]) or distant stage (metastatic) cancer (HR = 1.1 [95% CI 0.79 to 1.7]) (71). Raji et al (2013) found that approximately 33% of patients with a dementia diagnosis died within 6 months of a cancer diagnosis, while the proportion was 8.5% for patients without dementia (79). The difference varied by cancer types: prostate: 29% vs 4.3%; breast, 25% vs 5.1%; and colon, 45% vs 20%. In addition, the authors found that cancer stage at diagnosis explained 16% and 14% of the excess all-cause mortality in breast and colon cancer (both *P* < .001), respectively, but not in prostate cancer (79).

In addition to the 21 studies on mortality, three studies reported the median survival time for patients with cancer and dementia [mixed cancer types, 23 months (cognitive impairment) vs 73 months (no cognitive impairment), *p* < .001 (62); HR+ breast, 20 months for median disease free survival (DFS) and 23 months for overall survival (OS) vs 50 months for median DFS and 57 months for OS (62); triple negative breast, 5.3 months for median DFS and 14 months for OS vs 39 months for median DFS and 50 months for OS (62); solid tumors, 30 months vs 56 months (63)] and one reported the mean survival time for patients with cancer and dementia [stage III colon cancer, 57% that of patients without dementia (67)].

## Discussion

The primary aim of this mixed studies review was to understand cancer treatment decision-making practices in the context of dementia and how it affects the type of treatment patients receive and their subsequent outcomes. We extracted data from 55 recent studies to provide both qualitative and quantitative syntheses on several key themes including treatment decision-making, receipt of cancer treatment, and all-cause and cancer-specific mortality. Our mixed studies review includes the largest number of studies compiled thus far describing the impact of dementia on cancer treatment decision-making, treatment, and mortality. However, less than half of the papers in this review were grouped under the treatment decision-making theme (n = 22). While our findings corroborate existing knowledge about patients with both cancer and dementia in general, this mixed studies review identified new gaps in the existing knowledge base that could improve cancer treatment decision making from the perspectives of clinicians, patients, their families and/or caregivers. We found that dementia complicated cancer treatment decision-making in several ways: 1) dementia impairs decision-making capacity and the ability of the patients to provide informed consent for treatment; 2) communication difficulties may exist among the patients, family, caregivers, and health-care professionals; and 3) there is a lack of clear guidelines tailored for this unique population.

The coexistence of dementia and cancer imposes extra challenges on patients, caregivers, and clinicians in their decision-making than either cancer alone or dementia alone does. Recognizing the coexistence of cancer and dementia will improve earlier recognition of symptom burden and future development of diagnostic and/or therapeutic interventions. Dementia is a progressive disease that makes it challenging to assess for pain and discomfort (31,58,59,62). In addition, the capacity to weigh the risks and benefits for different treatments is important to provide informed consent and ultimately make decisions. All types of dementia, such as Alzheimer’s disease, Lewy body dementia, vascular dementia, and frontotemporal dementia, can impair the capacity to make decisions (82). It is often the clinician’s responsibility to assess whether a patient with suspected dementia has decision-making capacity. In cases where patients may not have caregivers or any legal surrogate, assessing for decisional capacity is challenging. Our review found that the health needs of those with both cancer and dementia are not often known or met, which complicates the process of determining what treatment is appropriate. Consequently, our findings demonstrated that caregivers and families relay health information and assist in decision-making for patients who are unable to do so themselves. Consistent with suggestions from one previous review (6), we recommend that future guidelines to incorporate caregivers in the evaluation and management of patients both cancer and dementia, as this could be of great benefit to oncologists.

Guidelines that outline how to identify and address dementia could aid oncologists in the treatment decision-making process. The National Comprehensive Cancer Network (NCCN) (83) and the American Society of Clinical Oncology (ASCO) (84) provide information to aide oncologists in identifying dementia. The NCCN’s guidelines lay out the assessment of cognitive function by listing conditions such as mild cognitive impairment, dementia, and delirium and expand on their definitions, features, differential diagnoses, screening tools, and recommendations for further evaluation. They suggest that older adults with dementia or any form of cognitive impairment can benefit from a referral to a geriatrician prior to cancer treatment to “develop a coordinated plan of care and/or manage geriatric syndromes that could jeopardize outcomes of cancer treatment (83).” The ASCO also provides resources on geriatric oncology for clinicians, such as trends and data on cancer and aging, geriatric assessment screening tools, and chemotherapy toxicity prediction tools (84). Given the projected increase in patients with cancer and dementia, oncology guidelines must incorporate the management of patients with dementia. Specific guidelines are necessary to improve consistency amongst clinicians when treating patients with similar conditions, which this mixed studies review found is currently lacking.

The literature supports facilitation of shared decision-making with an emphasis on open and clear communication 85–88). Those involved in making decisions regarding cancer treatment should be encouraged to voice their opinions or questions. McWilliams et al. (2018) state the impact of dementia on cancer treatment needs to be understood and accessible by caregivers (35). Poor communication is a barrier to the receipt of appropriate care (89,90). We examined composition of cancer care teams, particularly the involvement of geriatricians, which may also impact decision-making. Among 7 out 55 studies, geriatrician specialists were directly involved in the management of older cancer patients with or without dementia (27,29,43,52,55,74,75). All 7 studies support the notion that geriatricians play a key role in prioritizing the health needs of these patients, understanding their strengths and weaknesses, and optimizing their cancer treatment, whether curative or supportive. In addition, Flood et al. found that an Oncology-Acute Care for Elders (OACE) Unit interdisciplinary team that included a geriatrician was able to identify geriatric syndromes like dementia and/or delirium among older cancer patients (29). The authors propose that more studies are needed to compare outcomes of hospitalized patients with cancer receiving an OACE intervention with those patients receiving usual care (29). Taken together, we recommend incorporating a multidisciplinary geriatric oncology team from the beginning and additional training on geriatric conditions for oncologists may help with the processing, planning, and delivery of care and treatment. Future studies should emphasize the role of communication and care management using multidisciplinary care teams.

The overall trends among health care professionals and caregivers regarding treatment preference supported less aggressive treatment measures and enhanced focus on improving quality of life through hospice or palliative care. Resonating with this, our findings indicate that patients with comorbid cancer and dementia diagnoses are less likely to receive curative treatment and more likely not be treated at all or receive palliative or hospice care than patients with cancer only. Factors including comorbid conditions, like dementia, and low socioeconomic status are associated with late cancer stage at diagnosis, which may limit treatment options and lead to worse survival (64). Fearing that dementia patients lack an understanding of complicated treatment regiments, have impaired ability to withstand and benefit from treatment, or lack a patient advocate are some of the reasons behind clinicians’ choice of prescribing less or no treatment for dementia patients with cancer (27,54). The competing risk of mortality also plays an important role in treatment decision-making and the survival of cancer patients with dementia (27,34,39,51,53,54,61,64,69). The estimated median survival time from onset of dementia was 3.3-11.7 years (91), while the median survival time after cancer diagnosis depends largely on stage and type of cancer. Given the limited life expectancy for dementia patients and potential complications of treatment, clinicians and patient caregivers may opt to prioritize quality of life (27,53,54,61,64,76). In addition, clinicians may hold different opinions on the most appropriate cancer treatment to provide or forgo for patients with dementia.

Given older patients’ potentially limited ability to receive aggressive cancer treatment, we expected to find a larger number of studies on the utilization of palliative care or hospice among those with dementia and cancer. Instead, we found that most articles studied patients with either dementia or cancer, but not those with simultaneously both diagnoses. Palliative care and hospice provide supportive medical care—hospice is usually reserved for terminally ill patients, while palliative care can be provided during any stage of a serious life-limiting illness or treatment phase. Even in the absence of a diagnosis of cancer, patients with dementia are less likely to be referred to palliative care and often have pain that is not treated compared to patients without dementia (92). If involved early, palliative care teams may be able to identify and manage symptoms related to both cancer and dementia in patients who are receiving or planning to receive other treatments for cancer, thereby easing some of the burden of having both conditions. Thus, there is a need for further research on the use of palliative treatment to support patients with comorbid cancer and dementia.

While demographic factors varied between patient populations, 19 of 21 papers consistently found higher mortality rates for patients with both cancer and dementia compared to patients without dementia. While there is a need to better understand how dementia worsens survival for this population, this mixed studies review points to the role comorbid cancer and dementia plays in the difficulties regarding treatment decision-making. Furthermore, though patients with comorbid cancer and dementia experience worse outcomes than patients with either cancer or dementia alone, we found a lack of studies on the impact of the type of cancer treatment on survival among those with both cancer and dementia. We found one study that assessed whether any intervention at all was helpful in prolonging life for patients with stage III colon cancer (67). Their findings show that about 20% of worse survival in this population could be explained by the decreased odds of receiving chemotherapy (67). Future work should investigate which cancer-specific treatment types may improve survival in this cancer and dementia population.

## Limitations

While we included the largest number of studies compiled thus far in the current review, the available numbers of studies in each analysis is limited, thereby limiting power. In addition, there were large variations in the underlying study populations and methods of data collection among the reviewed studies, with most being quantitative studies and some qualitative and survey studies. Close to half of the studies were from a single institution or non-population-based data sources (n = 26), thereby limiting generalizability.

We were also limited by inconsistent definitions of dementia across the studies we reviewed, which may have resulted in an overestimation or underestimation of patients with true dementia. For instance, the terms “cognitive impairment” or “moderate or severe cognitive impairment” used to describe study participants could have been describing patients with true dementia, but the word “dementia” was not explicitly used. In those cases, a diagnosis of dementia may have been missed and resulted in an underestimation of patients with true dementia, thereby affecting the subsequent treatment decision-making (93).

While there are still gaps in the knowledge base, our conclusions are consistent with current practices and attitudes within the field. Our mixed studies review was limited by the few studies on patient populations with both cancer and dementia and even fewer studies assessing the impact of dementia on cancer outcomes. There was minimal research performed from the sole perspective of patients on decision making (28).

## Conclusion

This mixed studies review found that patients with cancer and dementia receive less aggressive cancer treatment than those without dementia and have higher all-cause mortality than those with either condition alone. Clinicians, patients, and their families and/or caregivers tended to opt for less aggressive or invasive treatment, yet decision-making for cancer treatment is still fraught with inconsistencies. Fragmented communication among health professionals, patients and their caregivers may be contributing factors. This review supports the development of concrete guidelines at the national level and the formation of multi-disciplinary teams for care-coordination. Future research should focus on the impact of dementia on cancer treatment decision-making, the appropriate utilization of palliative or hospice care, and multi-center data collection efforts for this growing dual-diagnosis population.

## Funding

The authors wish to acknowledge the support of the Mount Sinai Claude D. Pepper Older Americans Independence Center Grant 5P30AG028741 (K.D.) and 5P30AG028741-07 (B.L.) at the Icahn School of Medicine at Mount Sinai, the Biostatistics Shared Resource Facility (M.M.), Icahn School of Medicine at Mount Sinai, and NCI Cancer Center Support Grant P30 CA196521-01 (M.M.).

## Notes

**Role of the funders:** The funders had no role in the design of the study; the collection, analysis, and interpretation of the data; the writing of the manuscript; and the decision to submit the manuscript for publication.

**Disclosures:** The authors have no conflict of interest to disclose.

**Author contributions:** Conceptualization: BL, KD, YC. Data curation, Formal Analysis, and Methodology: YC, KD, BL, CG, MM. Supervision: BL, KD. Investigation: YC, BL, KD, CG, KAO, MM, NA, RMS, ET. Writing—original draft: YC, KD, BL. Writing—review & editing: KAO, NA, ET, RMS, MM, CG.

## Data Availability

No new data were generated or analyzed in support of this research.

## Supplementary Material

pkab002_Supplementary_Data
