# Supplementary material for: The Impact of Dementia on Cancer Treatment Decision-Making, Cancer Treatment, and Mortality: A Mixed Studies Review
Source: JNCI Cancer Spectr. 2021 Jan 27;5(3):pkab002. doi: 10.1093/jncics/pkab002 (PMC8152697; doi:10.1093/jncics/pkab002)
Supplement: pkab002_Supplementary_Data [file pkab002_supplementary_data.pdf]

Supplementary Table 1. ICD Dementia Ascertainment Codes

First author, Publication Year	ICD Dementia Ascertainment	Codes	Notes
Chen, 2015	ICD9	290-290.9	--
Chen, 2017	ICD9	290.40 to 290.43, 331.0, 331.1, 331.11, 331.19, 331.82, 046.11, 046.19, 292.82, 333.4, 290.10 to 290.13, 290.0, 290.20, 290.21, 290.3, 331.2, 331.9, 290.8, 290.9, 294.10, 294.11, 294.20, 294.21, 294.1, 294.8, and 797	331.83 for mild cognitive impairment
Neuman, 2013a	ICD9	331.0-331.1, 331.11, 331.19, 331.7, 290.0, 290.10-290.13, 290.20-290.21, 290.3, 290.40-290.43, 294.0, 294.1, 294.10, 294.11, 294.8, and 797	--
Raji, 2008	ICD9	331.0 to 331.2, 331.7, 290.0, 290.1, 290.10 to 290.13, 290.20, 290.21, 290.3, 290.40 to 290.43, 294.0, 294.1, 294.8, and 797	--
Neuman, 2013b	ICD9	331.0 to 331.1, 331.11, 331.19, 331.7, 290.0, 290.10 to 290.13, 290.20 to 290.21, 290.3, 290.40 to 290.43, 294.0, 294.1, 294.10, 294.11, 294.8, and 797.15	--
Patnaik, 2011	ICD9	Not reported	--
Lee, 2018	ICD9	290, 294.1, 331.0	--
Baillargeon, 2011	ICD9	331.0–331.2, 331.7, 290.0, 290.1, 290.10–290.13, 290.20, 290.21, 290.3, 290.40–290.43, 294.0, 294.1, 294.8, 797	--
Kedia, 2017	ICD9	290.x, 294.0, 294.1x, 294.2x, 294.8x, 331.0, and 331.82	--

Bradley, 2008	ICD9	331.0, 331.x, 290.0, and 797	
Gorin, 2005	ICD9	331.0, 290.0, 290.1, 290.2, 290.3, and 797	Diagnoses of AD occurring up to 6 months after cancer diagnosis were included
Gupta, 2004	ICD9	290 codes, 797, 331.0 codes, 331.2, 331.4, 331.89, 331.9	--
Zaorsky, 2017	ICD9/10	Not reported	--
Saffore, 2018	ICD9/10	290, 290.1, 290.11, 290.12, 290.13, 290.2, 290.21, 290.3, 290.4, 290.41, 290.42, 290.43, 291.2, 292.82, 294.1, 294.11, 294.2, 294.21, 331.1, 331.19, 331.82, F01.50, F01.51, F02.80, F02.81, F03.90, F03.91, F10.97	331.83 and G31.84 for mild cognitive impairment
Legler, 2011	ICD9	331.0 to 331.2, 331.7, 290.0, 290.1, 290.10 to 290.13, 290.20, 290.21, 290.3, 290.40 to 290.43, 294.0, 294.1, 294.8, and 797	--
Monroe, 2012	ICD9	331.0, 290.4, 331.82, or some combination thereof	--
Monroe, 2013	ICD9	331.0, 290.4, 331.82, or some combination thereof	--
Ording, 2013	ICD8/10	Not reported	--
Dening, 2016	ICD10	Not reported	--
Chang, 2014	ICD10	F00–03	--

Mohammadi, 2015	ICD10	F00-F03, F051, G30, G311	--
Morin, 2016	ICD10	F00-F03, F051, G30, G311	--

Supplementary Table 2. ICD9 and ICD10 Codes frequency

ICD Code	No. of studies
ICD9 Codes	
46.1x	1
290.x	15
291.x	1
292.x	2
294.x	9
331.x	14
333.x	1
797	9
ICD10 Codes	
F00.x	3
F01	4
G311	2
G30.x	2
